# Learning to teach with patients and caregivers: a focused ethnography

**DOI:** 10.1186/s12909-024-05197-5

**Published:** 2024-03-03

**Authors:** Sara Alberti, Valeria Vannini, Luca Ghirotto, Loris Bonetti, Sergio Rovesti, Paola Ferri

**Affiliations:** 1 Department of Biomedical, Metabolic and Neural Sciences, University of Modena and Reggio Emilia, str. Giuseppe Campi n° 287, Modena, 41125 Italy; 2 Qualitative Research Unit, Azienda USL-IRCCS di Reggio Emilia, viale Umberto I, Reggio Emilia, 42123 Italy; 3 Nursing Research Competence Centre, Ente Ospedaliero Cantonale, str. Officina n°3, Bellinzona, 6500 Switzerland; 4 Department of Business Economics, Health and Social Care, University of Applied Sciences and Arts of Southern Switzerland, str. Violino n°11, Manno, 6928 Switzerland

**Keywords:** Healthcare professional education, Patient and caregiver participation, Narration, Emotions, Interprofessional relations, Qualitative research

## Abstract

**Background:**

Little is known about what happens when patients and caregivers are involved in an academic setting as co-teachers and how healthcare professionals approach a new model of partnership-based teaching. This study aimed to explore the learning and behavioural patterns of a group of healthcare professionals who were learning to teach with patients and caregivers as co-teachers in a post-graduate course.

**Methods:**

A focused ethnographic study involving 11 health professionals was conducted. Data were collected through participatory observation during the course, individual semi-structured interviews, and a follow-up focus group. Taxonomic analysis was performed.

**Results:**

Three categories were identified: ‘group’, ‘role of narration’ and ‘applying co-teaching with patients and caregivers ’. Specifically, heterogeneity, absence of hierarchies, and balanced relationships characterised the group dynamic and promoted partnership. Narration played a key role both in learning and in healthcare professionals’ relationship with patients and caregivers and promoted emotional skills and self-awareness. Project planning and lessons simulations were essential aspects of the implementation process.

**Conclusions:**

This focused ethnography helped further understanding of the context of a specific project involving patients and caregivers as co-teachers in healthcare professional education. The development of emotional skills and self-awareness are the main learning patterns of co-teaching, and interprofessionalism and balanced relationships are the basis of the behavioural patterns. These patterns facilitated the involvement of patients and caregivers in health education.

**Supplementary Information:**

The online version contains supplementary material available at 10.1186/s12909-024-05197-5.

## Background

Recent years have seen a growing interest in involving patients and caregivers in the education process of healthcare professionals. This involvement is associated with promoting the relational mechanisms that foster patient-centred outcomes [1, 2]. The heterogeneity of educational activities that involve patients and caregivers has been a common focus of several reviews, which have demonstrated that patient and caregivers’ involvement is a complex educational intervention occurring in a social environment containing many stakeholders [3–9]. Patients involved are individuals with significant experience of illness or healthcare, and caregivers are carers who regularly provide care for a child, or a sick, elderly, or disabled person. Both groups utilize the skills acquired from their experiences and specific training to offer an integrated perspective in education and carry out educational interventions. They are involved in educational programs that focus on specific clinical condition such as mental health, chronic care, older adult care, paediatric care, disability and cancer, as well as organizational, communication and relational aspects of care [2, 8]. A recent review has described the multifaceted components of this educational intervention and concluded that more work is required to identify the mechanism through which patient involvement enhances learning and to explore what involvement within the education community means for both faculty and patients [10]. Furthermore, the effects on staff and patients as well as students have to be considered when evaluating educational programs that involve patients [11]. Patient and caregivers’ involvement in education has been associated with increased understanding of the importance of patient-centredness and communication, building and improving skills that help patients make informed decisions, and exhibiting appropriate professional behaviours and relationships demonstrating respect and compassion [7]. A randomised controlled trial showed that an educational intervention co-conducted by two patients (a 70- year-old woman with amyloidosis and a 40-year-old woman with previous breast cancer) and nursing teachers compared to an intervention conducted by the nursing teachers alone was more effective in increasing empathy levels in nursing students [12].

However, little is known about what happens when patients and caregivers enter an academic setting as teachers. Involving patients in education means changing the traditional educational paradigm, in which only healthcare professionals act as teachers, versus an alternative based on partnership between healthcare professionals and patients as respective experts in disciplinary and experiential knowledge. The latter can be complicated to implement; nevertheless, few studies have examined how the role of teachers and context change. In their review, de Groot et al. found two prominent teacher roles in educational interventions that involved patients: carer and assessor [1]. The first role implies that the teacher is not primarily the ‘expert who will give all the answers’ but rather someone who cares for learners and recognises that interactions with severely ill people for the first time might be challenging. In contrast to this role, wherein teachers feel their primary responsibility is to support learners, is that of assessor, in which the teacher evaluates whether students have made sufficient progress and are ‘good enough’.

Moreover, some studies have found negative attitudes from healthcare-professional teachers toward patient involvement in education; for example, patients’ views can sometimes conflict with those of healthcare professionals, causing the latter to worry their own expertise could be devalued [13]. In most studies patients and caregivers are involved together and differences in results are not presented, however a qualitative study, which presents the perspective of two caregivers involved in education and the perspective of academics, reports how the latter were worried about what they perceived as a possible shift in the balance of power. Their chief concern was who would have the final say on which students should be offered a place if they disagreed with carers. Despite their initial doubts and reservations, once the academics had a chance to work with them they reflected on the experience positively [14]. A recent ethnographic study reported that transforming the ‘caregiver–patient’ relationship into a ‘colleague–colleague’ relationship generated identity upheavals among healthcare professionals [15]. When called to work with patients or caregivers outside of a simple therapeutic relationship, healthcare professionals may feel tension between their identities as caregivers and as colleagues. Identity tensions included competing ideals of the ‘good professional’, challenges to the permeability of the ‘patient’ and ‘professional’ categories, the interweaving of symbols associated with one or the other of these identities, and the inner balance between the roles of caregiver and colleague.

As a complex intervention, implementing patient involvement in Healthcare Professionals Education (HPE) requires an understanding of the context in which the practice is to be used. For example, how teachers work together, leadership and organisational support for new practices, school culture, faculty resources, and other factors can influence implementation. Further, what works in one setting and course and with one group of students may not work in another. Implementation studies can be used to explore these factors; they take place in real-world settings, so researchers’ task is to identify potentially relevant variables that might influence the outcomes and to incorporate these into the study, often as research questions. Examples of these variables are teachers’ shared beliefs, learning patterns, and behaviours [16].

### Aim and research question

This study is part of a research project whose overall objective is to implement patients’ and caregivers’ involvement in HPE as a complex educational intervention. Accordingly, this study aimed to describe the experiences of healthcare professionals participating in a post-graduate teaching course that included patients and caregivers as co-teachers. The following research question was set: What are the shared beliefs and learning and behaviour patterns of healthcare professionals in a post-graduate course regarding patients’ and caregivers’ involvement in HPE?

## Methods

### Theoretical framework

Focused ethnography, guided by established guidelines for health research, was employed to explore the culture of a small group of healthcare professionals engaged in patient and caregiver education [17]. This validated research approach involves direct observation of personal experiences and behaviors within their natural context. The study delved into changes in thinking and behaviors among healthcare professionals within a specific dynamic setting, aligning with transformative learning theory. In HPE, transformative learning theory, as highlighted by Ryan et al. (2022), is increasingly employed as a framework to understand how healthcare professionals learn in ways that promote fundamentally new ways of thinking and behaving [18]. This theory harmonizes with the ethnographic approach. By comprehending group learning patterns, values, and beliefs through ethnography, the study aims to support the implementation of effective educational interventions involving patients and caregivers as teachers [19, 20].

### Setting and participants

The study was conducted at the University of Modena and Reggio Emilia in Italy as part of a research project promoted by the EduCare Lab, an interdepartmental laboratory aimed to involve patients and caregivers in HPE [21].

To promote the implementation of the new teaching methodology, in the 2021–2022 academic year, the post-graduate course ‘Didactic methodology for teaching with patients and caregivers as teachers’ was conducted. Its objectives were to teach patients, caregivers, and healthcare professionals how to collaborate in educational interventions, called ‘lessons-in-tandem’. The course lasted from December 2021 to April 2022 and it was structured in 5 modules. Additional file 1 summarises the course syllabus.

The course was attended by 9 patients, 2 caregivers and 11 healthcare professionals (one researcher [SA] was included in the latter). All healthcare professionals participating in the post-graduate course were included (*n* = 11). Table 1 shows participants’ demographic and professional characteristics.

**Table 1:** Participant characteristics

Demographic and professional characteristics	*n*
Gender	
Male	3
Female	8
Age (years)	
25–34	3
35–44	2
45–54	1
55–64	3
+65	1
Profession	
Physician	3
Nurse	5
Physiotherapist	1
Midwife	1
Psychotherapist	1
Professional experience (years)	
<5	2
5–10	1
11–20	3
21–30	0
>30	5
Main professional field	
Clinical	5
Education	5
Teaching experience (years)	
None	1
<5	3
5–10	1
11–20	1
21–30	3
>30	2
Teaching experience with patient as teacher	
Yes	5
No	5

### Research team and reflexivity

In the context of this focused ethnography, SA assumed the role of principal investigator, leveraging her background as a nurse and a Ph.D. student in clinical and experimental medicine. Actively engaged in the EduCare Lab and a broader research initiative on patient involvement in education, SA enrolled in the post-graduate course associated with this study. Her intent was to delve deeply into the intricacies of patient and caregiver involvement in education, enhance her proficiency in this domain, and consequently, refine the execution of a multifaceted educational intervention. Employing participatory observation, SA, situated as an insider within the milieu under scrutiny, recognized the potential impact of her insider position on research dynamics, including researcher contributions, methodological choices, and data interpretation.

SA’s insider perspective aligns with a cultural relativist framework, a stance posited for its manifold advantages, as elucidated by Holmes et al. [22]. These advantages encompass facilitated access to the studied setting, the formulation of more incisive queries, heightened trust fostering candid responses, the capacity to generate a more authentic and detailed portrayal, and a nuanced understanding of the language employed. However, the concomitant risk lies in potential inherent bias and an inadvertent inability to introduce an external perspective to the research process, a facet acknowledged by SA, who is a novice researcher in the realm of focused ethnography.

To address potential biases, SA adopted two strategic measures: reflexivity and the involvement of external researchers unaffiliated with the EduCare Lab and the post-graduate course. Engaging in self-reflection, SA embraced a reflexive approach to delineate her positionality, elucidate expectations, uncover assumptions, and scrutinize conscious and unconscious reactions to the context, participants, and data [23]. This introspective process was documented in a reflective ethnographic diary, diligently maintained throughout and after the course, alongside contemporaneous field notes. The diary encapsulated SA’s personal experiences, thoughts, emotions, and expectations. During the analysis phase, this reflective record became a focal point for discussions within the research team, comprising LG, VV, LB, and SR. Importantly, these team members, distinct from course participants and unaffiliated with the EduCare Lab, provided an external perspective crucial for scrutinizing the potential influence of SA’s positionality on domains and emerging categories.

Within this team, LG, an adept qualitative researcher, brought prior experience in focused ethnography to the table, complementing the diverse expertise within the group. Notably, PF, a member of the EduCare Lab, did not partake in the course, further diversifying the perspectives involved in the study. This collaborative approach, incorporating both reflexive practices and external insights, aimed to mitigate biases and enhance the rigor of SA’s exploration into patient and caregiver involvement in education within the studied context.

### Data collection

Data collection began in December 2021 at the same time as the course and ended in November 2022 with a follow-up focus group. The data sources included the available course documentation (i.e. syllabus, bibliographic material, forms completed during the course), field notes, individual semi-structured interviews, and a focus group. The field notes contained observations by the researcher and conversations with the participants, useful for capturing latent aspects of learning, the relational dynamics, verbal and non-verbal behaviors of the trainees during the course, and to understand their point of view and how they experienced the training. The observation protocol was based on the research question in line with the ethnographic approach [24]; 42 h of observation were performed. Semi-structured and audio-recorded interviews were conducted during the weeks following the end of the course; they lasted an average of 60 min. A guiding question approach was used, which was modified several times during the data collection phase based on the first analysis of the interviews already conducted on the professional background. However, in every case, a non-directive approach was consistently employed to avoid influencing or steering the responses too much.

Seven months after the end of the course, a focus group was conducted and audio-recorded, the script for which was based on the analysis of the data collected. VV conducted all interviews and the focus group. The first draft of the interview and focus group guides are shown in Additional files 2 and 3, respectively.

### Data analysis

**Fig. 1:**
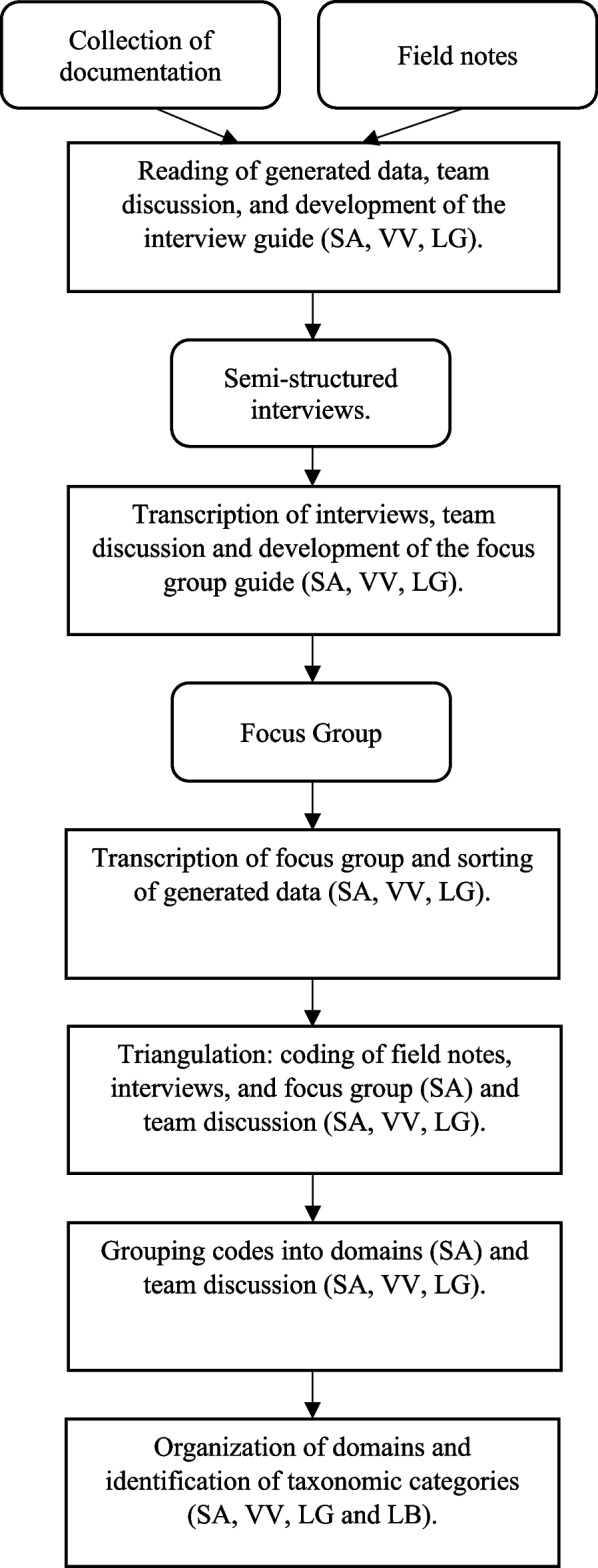
Analysis process

Discerning participants’ behaviour and learning patterns and a framework of their values and beliefs was achieved through description, interpretation, and critical analysis using taxonomic analysis process as recommended by Robinson [25]; this approach had been previously used by LG, a research team member, as a qualitative research expert [26]. The ethnographic research process began by gathering descriptive data and identifying them according to domains. Following domain analysis, taxonomic analysis was conducted. It is an inductive process. The phases of the analytical process are described below and showed in the Fig. 1.

1. Sorting of collected materials: Course documentation, field notes, interviews, and focus group verbatim transcripts were extensively reviewed by SA, VV, and LG throughout the data collection process.
2. Coding of field notes, interviews, and focus group and triangulation: SA organized codes and formatted them into tables according to data type. Then, she wrote descriptive accounts of the data from different sources. Through a meeting, SA, VV, and LG reached an agreement about the main similarities and differences.
3. Domain analysis: SA grouped codes into domains. Domains correspond to categories of meaning that enclose patterns of learning, behaviors, beliefs, and values; through meetings, SA, VV, and LG compared domains with raw data and descriptive accounts. These helped researchers triangulate data to establish trustworthiness.
4. Taxonomic analysis: This consisted of the process of determining the organization of domains until categories arose; taxonomic categories were determined through discussion and agreement of SA, VV, and LG. Another researcher involved in this phase was LB.

## Results

Three categories were identified through data analysis data analysis: ‘Group’, ‘Role of narration’ and ‘Applying co-teaching with patients and caregivers’.

### Group

This category includes patterns of behaviour, learning, values, and beliefs from the group of participants in the post-graduate course relevant to teaching with patients and caregivers as teachers. It includes two domains: ‘Heterogeneity’ and ‘Relationship-building’.

#### Heterogeneity

 A participant emphasized that a balanced number of patients and healthcare professionals with experiences in different clinical areas allowed the focus to shift from pathology to illness, that is, to the bio-psychosocial aspects (see Table 2: 1.a). Interprofessionalism along with age differences then characterized the group. This was perceived as an added value because diverse viewpoints enriched discussions, promoting a broader view of reality and increasing awareness (1.b). Furthermore, the heterogeneity along with the absence of hierarchies directed efforts towards the shared goal of becoming teachers together to humanize care (1.c), contributing to the development of skills such as empathy and patient-centeredness, and thus becoming better healthcare professionals (1.d).

**Table 2:** Quotes from participants and field notes for the ‘Heterogeneity’ domain

Identification code	Participant quotes and field notes
1.a	*There was a balance between the number of professionals and patients, and this is positive. I really liked the fact that the professionals belonged to different disciplines and were of different ages and educational backgrounds because I think it was a very enriching element…it was an opportunity for a very good discussion and to also create a collaboration with different points of view, and I really liked that the patients told stories about different pathologies…the idea is to focus on one's suffering in general, here it is, regardless of the pathology.* (INT_08)
1.b	*Certainly, the heterogeneity of the group and the absence of hierarchies allowed us to gain a broader vision, from more points of view; thus, to better understand the reality. The more points of view, the more roles I interface with, the more I become aware of my role in that context in relation to others. This was the collaborative learning we experienced.* (INT_01)
1.c	*It was a richness …common elements emerged spontaneously in the group…the final objective was to humanise medicine, which does not care about people, a little.* (INT_06)
1.d	*One participant during the lesson described himself as a curious person at the beginning of the course and now as a person writing his story, and in a year's time, he sees himself as a person who, through self-narration and discussion with others, has achieved a certain degree of freedom and awareness. Another participant stated that he sees himself as a person who is increasing his empathy and patient-centred skills. Another professional said that he sees himself as a better health professional in the future through this group.* (FN)
l.e	*Often, the young healthcare professionals and caregivers moved in and out of the activity and during social moments together. Their attitude changed when addressing the elderly; they were more formal in their behavior and communication. Furthermore, they were often seen as a subgroup frequently identified with the term "the young." They were mostly considered inexperienced, with little experience, both in a negative sense and positively as a driver of change.* (FN)

*Abbreviations*: *INT* Interview, *FG* Focus group, *FN* Field notes

The different ages of the participants led to the creation of a subgroup nicknamed “the young”, labeled as inexperienced yet agents of change. However, this did not hinder the group building and the execution of the activities (1.e).

Excerpts from interviews and participant focus groups, along with field notes related to this domain, are provided in Table 2.

#### Relationship-building

Feeling part of a peer group promoted the active and balanced participation of all members; indeed, field observations indicate that healthcare professionals and patients spontaneously contributed to discussions (Table 3: 1.f); furthermore, active communication between patients and healthcare professionals was perceived as rewarding despite linguistic heterogeneity (1.g). Such relationship-building behaviours facilitated group cohesion. Closely related to this aspect is the fact that face-to-face lectures were crucial for getting to know each other and thus for group-building and achieving the course learning objectives (1.h); also convivial moments outside of the course, such as dining together and attending the theatre, were important (1.i).

**Table 3:** Quotes from participants and field notes for the ‘Relationship-building’ domain

Identification code	Participant quotes and field notes
1.f	*Patients and professionals all spontaneously add to the discussion without distinction of roles.* (FN)
1.g	*We created an important communication network…It was also important to listen and to listen eh…really what on the other hand is transmitted not only with verbal language but also non-verbal language, which is another thing I’m very sensitive to.* (INT_02)
1.h	*We did some face-to-face lessons, and I really enjoyed it because I got to know both patients and professionals better and work better in a group*. *This was essential to learning to teach* together. (INT_01)
1.i	*There were some convivial moments, such as dining together and going to the theatre…that help to create a group dynamic…this was a very good idea from the organisers…let's say winning.* (INT_04)
1.l	*I have already done a workshop programme…I’m collaborating with colleagues to put the workshops into practice…and the first year, we wanted to focus on the caregiver […] the second year, we wanted to focus instead on a person with a chronic health problem […] and the third year […] we titled 'the end of a journey'.* (INT_10)
1.m	*I and other colleagues will begin this project very soon…the design was complex, and I must say challenging…we will see how it goes.* (FG_01)

*Abbreviations*: *INT* Interview, *FG* Focus group, *FN* Field notes

The group cohesion is also demonstrated by the fact that it continued to work on implementing the new teaching methodology after the course ended. In fact, during the interviews, two healthcare professionals mentioned that they had already discussed the planning of this project, and six months later, during the focus group, the same professionals stated that the project was in the start-up phase and that they were still working together (1.l, 1.m).

Table 3 displays excerpts from interviews and participant focus groups, along with field notes related to this domain.

### Role of narration

During Module III (Additional file 1), healthcare professionals wrote down one of their experiences of illness, caregiving, or professional practice and shared it with the group. Significant learning and behavioural patterns, beliefs, and values stemmed from this experience. This category included three domains: ‘Self-narration as an emotional and cognitive learning mechanism’, ‘Healthcare professionals’ difficulty telling their story’, and ‘Perceptions of narration during co-teaching with patients and caregivers’.

#### Self-narration as an emotional and cognitive learning mechanism

Self-narration has been defined as a fundamental step and key to learning to teach with patients and caregivers as co-teachers because it promote self-awareness and emotional skills (see Table 4: 2.a,). In this study, it helped participants understand their own stories and achieve a balance within themselves and with their emotions (2.b). In addition, during the focus group, one participant stated that self-narration motivated him and encouraged him to actively participate in the activities of the course because there were emotions behind it (2.c). In this sense, self-knowledge was fundamental to interfacing with patients, understanding their needs, and humanising care. During the postgraduate course, the participants learnt to analyse the humanising dimensions contained in the narrative according to a specific theoretical framework; in this way learning activity shifted to the cognitive level (2.d).  Moreover, sharing narratives with the group led healthcare professionals to understand the common elements between the stories of patients and those of healthcare professionals, as well as the awareness that both are people (2.e).

**Table 4:** Quotes from participants and field notes for the ‘Self-narration as an emotional and cognitive learning mechanism’ domain

Identification code	Participant quotes and field notes
2.a	*To understand the experience of patients and caregivers and learn to teach with them, we must first know ourselves…and the only way is to know ourselves by working on ourselves…personal growth goes hand in hand with professional growth.* S*elf-narrative in this course helped make this fundamental transition.* (FG_01)
2.b	*Telling the caregiver's personal story…it was an important exercise in the sense that it helped me better understand how I am in relation to my story and my emotions…it helped me a lot to rebalance some parts that were imbalanced.* (INT_02)
2.c	*It’s an excellent tool for giving motivation…it promotes active participation because there are emotions behind it, and one is personally involved…it’s what has most contributed to developing deeper thoughts on the doctor–patient relationship.* (FG_05)
2.d	*The participants in the course were divided into heterogeneous groups with the aim of analyzing a story and identifying humanizing dimensions according to a specific framework. During this activity, there was no distinction between patients and professionals, and the focus of the discussion shifted to the facts of the story rather than the emotional experience*.(FN)
2.e	*The narration of oneself was to rediscover that there’s no difference between me and the patient…we are two human beings…there is the rediscovery of how vulnerable I am and how vulnerable the other is…otherwise, the risk is to see the patient as a body to be fixed.* (FG_09)

*Abbreviations*: *INT* Interview, *FG* Focus group, *FN* Field notes

Table 4 presents quotes from participants and field notes to illustrate this domain.

#### Healthcare professionals’ difficulty in telling their stories

Nevertheless, narration was difficult for healthcare professionals. They had trouble telling their stories because, unlike patients, they are not used to reflecting on themselves or managing any arising or connected emotions (see Table 5: 2.f). In fact, when choosing what experience to tell, healthcare professionals stated that they considered their own ability to manage that experience emotionally (2.g). Another perceived difficulty in narration was related to being in a group setting: the fact that this activity took place at the beginning of the course when the participants did not yet know each other was an obstacle (2.j). Consequently, sharing the story with the group became an emotionally intense moment (2.k), and this emotional engagement was also positively perceived after six months (2.l).

**Table 5:** Quotes from participants and field notes for the ‘Healthcare professionals’ difficulty in telling their stories domain’

Identification code	Participant quotes and field notes
2.f	*It was also difficult because for the first time, I wrote something about my experience highlighting my emotions, as well.* (INT_02)
2.g	*In my opinion, not enough work has been done on professionals’ narratives…we hardly ever reflect on our own experiences, our own emotions, and being able to tell them also allows patients to start learning to decentralise and thus also to put themselves in the head of the professional to grasp what the difficulties may be in the care pathway here.* (INT_09)
2.j	*It’s not easy to share such intense experiences with people you don't know, but then you think that if we’re all here, it’s because we want to help each other to learn to work together.* (INT_05)
2.k	*All the participants narrated their story in front of the group, holding the sheet of paper on which they had written it. Everyone was silent as they listened, and they were in an attentive attitude, the narrator's tone of voice varied, and there were often pauses, revealing the emotion in their storytelling*. (FN)
2.l	*The narratives are very important and emotionally very impactful because I’m still shaken now. (*INT_01)

*Abbreviations*: *INT* Interview, *FG* Focus group, *FN* Field notes

Excerpts from interviews and participant focus groups, along with field notes related to this domain, are provided in Table 5.

#### Perceptions of narration during co-teaching with patients and caregivers

Participants considered narration to be central in the context of the EduCare Lab (see Table 6: 2.m) and a useful tool for students to reflect on themselves (2.n). According to participants, teacher skills and planning were important for narration to be effective and to avoid any negative effects on students. Relatedly, in the interviews, healthcare professionals emphasised that time management of narration is important to prevent exposing students to excessive listening fatigue and suffering (2.o). The management of time and emotional involvement are aspects to consider when using storytelling as a teaching method. (2.p).

**Table 6:** Quotes from participants and field notes for the ‘Perceptions of narration during co-teaching with patients and caregivers’ domains

Identification code	Participant quotes and field notes
2.m	*Narration is a cornerstone of our system.* (FG_04)
2.n	*A participant during the lesson states: ‘Through the narration of my story, I acquire greater self-awareness, through my experience as a professional I can learn…that’s why it’s also useful for students…it’s a learning path, I learn myself, and I learn with others…plus, through narration, I bring a different point of view’.* (FN)
2.o	*It’s not how much time we expose people to a story but how much we think the people listening have the tools then to handle the pain inside here…so, this is the quality of listening, not the quantity of hours of listening.* (INT_09)
2.p	*Timing management and emotional involvement are important aspects of teaching through narration.* (INT_03)

*Abbreviations*: *INT* Interview, *FG* Focus group, *FN* Field notes

Raw data related to this domain, are provided in Table 6.

#### Applying co-teaching with patients and caregivers

This category encompasses healthcare professionals' view of teaching collaboratively with patients in their specific context, the learning mechanisms and behaviour of the lesson-in-tandem, and the implementation-directed behaviour identified from the data analyses. This category includes the domains ‘To start involving patients and caregivers in education’ and ‘Roles and relationships during lessons’.

#### To start involving patients and caregivers in education

According to participants’ points of view, involving patients as teachers responds to the learning need of patient-centred care and expressing empathy (see Table 7: 3.a). Patients and caregivers-as-teachers are not only recipients of care and experts in their illness or carers, but they also have experiential knowledge and teaching skills, especially in the use of narration (3.b). However, the patient’s state of health may be unstable, which may mean the way the course is conducted has to be modified (3.c). During the focus group, there was consensus that a lesson-in-tandem requires specific skills (3.d) and project planning, in which the healthcare professional and patient share learning objectives and how to use narration (3.e). Reflecting on implementation during the focus group, healthcare professionals agreed that involving patients in an academic setting was key for promoting interprofessional education in the healthcare field (3.f).

**Table 7:** Quotes from participants and field notes for the ‘To start involving patients and caregivers in education’ domain.

Identification code	Participant quotes and field notes
3.a	*The patient-teacher responds to the learning need of patient-centred care and is empathetic.* (INT_02)
3.b	*I thought the patient-teachers had knowledge that came from in-depth experience and were really good at revealing their stories.* (INT_07)
3.c	*Today's lesson was conducted online instead of in person as scheduled. We received communication from the teacher explaining that this decision was made to avoid putting at risk the already fragile health of the patients participating due to the increase in COVID-19 cases.* (FN)
3.d	*A healthcare-professional teacher must be trained in working with patients because lessons-in-tandem require specific skills.* (FG_01)
3.e	*We’re trained professionals, so we can develop projects, but we must also train the other teachers who carry out these projects.* (FG_09)
3.f	*In my opinion, the faculty of medicine and surgery, where there is a great need, is the priority…if we agree that the project must start and then develop from the medical aspects in synergy with other professions, in terms of interdisciplinary work, this is a key project.* (FG_10)

*Abbreviations*: *INT* Interview, *FG* Focus group, *FN* Field notes

Data from interviews, focus group and filed notes related to this domain, are provided in Table 7.

#### Roles and relationships during lessons

During Module V of the course, each healthcare professional was tasked with preparing and simulating a lesson in collaboration with patients and caregivers, called lesson-in-tandem, aimed at fostering partnership. All participants reported that the tandem-lesson simulation was an opportunity to interact with patients and caregivers frequently and significantly (see Table 8: 3.g, 3.j). During this activity, one healthcare professional compared his story with that of the patient, which allowed him to gain new awareness and overcome prejudice (3.k).

**Table 8:** Quotes from participants and field notes for the ‘Roles and relationships during lessons’ domain

Identification code	Participant quotes and field notes
3.g	*Preparing the lesson-in-tandem was a moment where I learnt a lot…because I was face-to-face with the patient and his story…in this way, it was very important to create the relationship.* (INT_05)
3.j	*[During the lesson-in-tandem,] we had synergy, we synchronised, in short, we built this lesson-in-tandem together, and it was very interesting.* (INT_02)
3.k	*It was amazing to read her story; first, it made me think of my own, it’s as if a part of her story was also in me. Then, for the tandem lesson, we reworked it to make the group understand the difficulty a person can have when returning home after hospitalisation; this made them overcome prejudices…which is what happened to me.* (INT_01)
3.l	*It struck me that the interaction between a healthcare-professional teacher and a patient is not obvious but requires skills, i.e. modalities known by both parties that can help them create a purposeful dialogue.* (FG_04)
3.m	*Patients are not only the ones who tell their story…but they teach…You, the healthcare professional who now knows the patients’ stories, should not use them as a tool to teach your lesson because it’s the patients who, through their stories, teach, and you can be a guide and help… (*INT_03)
3.n	*During the tandem lesson, several aspects were repeated: professionals presented the patients, placing them at the center of attention, managed the slides and the computer, intervened at the beginning and end of the lesson to introduce and summarize the transmitted content, and moderated the discussion.* (FN)

*Abbreviations*: *INT* Interview, *FG* Focus group, *FN* Field notess

The relationship between patient-teachers and healthcare-professional teachers required specific skills and partnership, as stated by one healthcare professional during the focus group (3.l). Three participants said they had designed the lesson together with the patient by analysing his/her story to identify the most significant parts and deciding together on the organisational aspects of the educational intervention. The basis of this behaviour is the belief that patients are not only the ones telling their own stories but also teachers with an active role. Similarly, healthcare professionals modified their traditional roles to that of facilitators (3.m) Indeed, during the simulation of the tandem lesson, they introduced the objectives and the activity, presenting the patient and immediately giving them the floor. They managed the bibliographic material and moderated the discussion by summarizing the transmitted content at the end (3.n).

Table 8 presents quotes from participants and field notes to illustrate this domain.

## Discussion

This focused ethnography investigated the shared beliefs and values, learning, and behavioural patterns of a group of healthcare professionals who were learning to teach with patients and caregivers.

The analysis showed that narration played a key role both in learning and in the relationship between healthcare professionals and patients/caregivers to teach together. As a learning mechanism, self-narration promotes self-awareness and emotional skills; this approach builds on reflective practice [27] and critical self-examination [28]. In this case, it allowed healthcare professionals to step back, review their stories, and recognise their thoughts, feelings, and emotions. While recognising the importance and value of these activities, they simultaneously described the process as difficult, because they were not used to self-reflection and managing emotions, which require narrative skills and a high degree of emotional intelligence [29, 30]. There is evidence that narrative approach in education strengthens healthcare professionals’ learning and boosts their professional and personal development [31]. Although there is a lack of studies evaluating these skills in healthcare professionals, it has been shown that writing exercises promote learning transformation, normalise emotions, create critical self-awareness, and provide a safe, non-judgemental space for students to reflect on their practice and learning [32, 33]. Time management and emotional impact should to be considered in the design of this educational activity, as excessive and prolonged exposure to human suffering can cause distress in healthcare professionals [34]. Our findings suggest this could also happen in education [35] and be an obstacle to its effectiveness [36]. Narration constitutes an interpersonal exchange between someone who tells a story and another who listens to it [37]; in the current study, it is defined as an exchange of perspectives between patients and healthcare professionals that make it possible to overcome prejudice and stereotypes and achieve the key aspect of a relationship with the patient. Thus, in line with the literature, narration in this context became a relational process that facilitates transformative learning [38].

The study's results highlight that the process of learning to teach alongside patients or caregivers constitutes a form of transformative learning [39]. This is underscored by the demand for professionals to possess the capacity to reassess and articulate their own ideas, beliefs, and constructs [18]. Self-narration, active engagement in the narratives of patients and caregivers, and the exchange of diverse perspectives play pivotal roles in fostering this transformative process. These elements contribute to the heightened awareness of shared humanity as a unifying factor between healthcare professionals and patients—a crucial realization in the journey toward partnership.

The tandem-lesson simulation further demonstrated the development of a meaningful relationship between practitioners and patients, characterized by shared decision-making and the absence of hierarchies. This observation aligns with a recent focused ethnography exploring identity changes in collaborative partnerships between patients and healthcare professionals [15]. Similarly, our study found that the caregiver–patient relationship transformed into one of colleague–colleague during the simulation, leading to identity upheaval and an internal balance between roles. Starting from narration, both rediscovered themselves as human beings with their own vulnerabilities. This helped eliminate hierarchy in the relationship and reconsider roles. However, unlike this previous study, healthcare professionals did not identify themselves in the roles of evaluators or caregivers but rather as facilitators, meaning those who facilitate the patient's role as a teacher[15]. Additionally, this study revealed that patient education as a teacher and the acquisition of educational skills are crucial aspects that facilitate the construction of the partnership and the absence of hierarchies in the relationship between educators. In line with the literature, we found the patient was seen as a person who had teaching skills but who also may have an unstable state of health, and this aspect should be considered in any HPE programme [40, 41].

A comparison and broadening of perspectives and attitudes of patient-centredness have been reported by several studies on educational interventions with patients as teachers [2, 42–45], and the current study’s results also suggest that the healthcare professionals who co-led lessons with patients achieved these outcomes.

Interprofessional education is considered a key factor in providing patient-centred care [46, 47]; the post-graduated in our study took place in an interprofessional education context, as it involved various healthcare professionals, patients, and caregivers with the goal of promoting partnership. The implementation of this new teaching method would therefore also promote interprofessionalism, which is realized not only in care but also in learning and education. For this to occur, interpersonal relations are important in the construction of social processes [48]; active communication and balanced relationships characterised this context and was facilitated by face-to-face teaching and informal socialising, which is consistent with past research [47].

Finally, in our study, we found that even six months after the course, the group remained active and continued to work on the implementation of this teaching methodology, emphasising the importance of project planning over several years and in an interprofessional context; these aspects are supported in the literature [49, 50].

There are some limitations. This study investigates the behavior and learning patterns of healthcare professionals rather than patients and caregivers involved. This choice was made because changing the paradigm of traditional education, which is based on the healthcare professional-teacher-student relationship, represents a challenge. Resistance to change among healthcare professionals is identified in the literature as a barrier [2]. However, exploring the perspectives of those receiving care is desirable to examine behavioural patterns of partnerships and gain a more comprehensive understanding of them. Therefore, studies that integrate various perspectives are encouraged. Closely connected to this aspect is the fact that participants were mostly nurses who volunteered for this study likely felt strongly about the topic and had more than 30 years of experience; therefore, their perceptions may not be representative of those of other faculty members and healthcare professionals.

Moreover, the researchers were all healthcare professionals; the diversity of backgrounds and different perspectives (inside and outside) contributed to mitigating biases and enhancing rigor. However, incorporating the patient's voice in research would be desirable.

Additionally, focused ethnography is context-specific [17, 51], and as a result, the findings of this study may not be generalizable to other contexts. Nevertheless, comparing the results with similar studies provides valuable insights for generating hypotheses in quantitative or mixed-methods research, such as investigating effectiveness in patient-centeredness, empathy, or interprofessionalism; this aspect in this study has been addressed partially.

## Conclusion

This focused ethnography helped further understanding of the context of a specific project involving patients and caregivers as teachers in HPE, providing useful insights to implementing effective educational interventions. Post-intervention, the healthcare professionals were more motivated to involve patients in HPE. Learning patterns were characterised by the development of emotional skills and self-awareness, and interprofessional relations and balanced relationships were the basis of behavioural patterns.

### Practical implications

The results of this study have demonstrated that, prior to implementing educational interventions involving students, healthcare professionals, patients, and caregivers, collaborative efforts are essential. This collaboration aims to cultivate emotional skills, self-awareness, and the establishment of effective partnerships. Self-narration and simulations of educational interventions play pivotal roles in facilitating this process. Moreover, this study helps the involved professionals and researchers gain awareness of the experience of learning to teach with patients and caregivers, allowing them to reflect on how to effectively promote the project; this is an advantage of focused ethnography [17, 51].

In addition, several aspects to consider in the implementation of the educational intervention have been identified. Project planning is as an essential phase of the implementation process, closely linked to the teacher's skills. Emotional skills and patient-centered care attitudes have been identified as the main learning outcomes for the students.

### Supplementary Information

**Additional file 1.** SyllabusT. his file contains the first draft of the syllabus of the post-graduate course ‘Didactic methodology for teaching with patients and caregivers as teachers’.

**Additional file 2.** Interview guide. This file contains the first draft of the semi-structured interview guiding questions.

**Additional file 3.** Focus Group guide. This file contains the first draft of the focus group guide.

## Data Availability

The datasets generated and/or analysed during the current study are not publicly available due individual privacy could be compromised but are available from the corresponding author on reasonable request.

## Abbreviations

HPE: Health Professional Education
INT: Interview
FG: Focus group
FN: Field notes

## References

[1] de Groot E, Schönrock-Adema J, Zwart D, Damoiseaux R, Van den Bogerd K, Diemers A, Grau Canét-Wittkampf C, Jaarsma D, Mol S, Bombeke K (2020). Learning from patients about patient-centredness: a realist review. BEME Guide 60 Med Teach.

[2] Alberti S, Ferri P, Ghirotto L, Bonetti L, Rovesti S, Vannini V, Jackson M, Rossi F, Caleffi D (2023). The patient involvement in nursing education: a mixed-methods systematic review. Nurse Educ Today.

[3] Towle A, Bainbridge L, Godolphin W, Katz A, Kline C, Lown B, Madularu I, Solomon P, Thistlethwaite J (2010). Active patient involvement in the education of health professionals. Med Educ.

[4] Scammell J (2015). Service-user involvement in nurse education. Br J Nurs.

[5] Suikkala A, Koskinen S, Leino-Kilpi H (2018). Patients’ involvement in nursing students’ clinical education: a scoping review. Int J Nurs Stud.

[6] Rowland P, Anderson M, Kumagai AK, Mcmillan S, Sandhu VK, Langlois S (2019). Patient involvement in health professionals’ education: a meta-narrative review. Adv Heal Sci Educ.

[7] Dijk SW, Duijzer EJ, Wienold M (2020). Role of active patient involvement in undergraduate medical education: a systematic review. BMJ Open.

[8] Gordon M, Gupta S, Thornton D, Reid M, Mallen E, Melling A (2020). Patient/service user involvement in medical education: a best evidence medical education (BEME) systematic review. BEME Guide 58 Med Teach.

[9] Soon YE, Murray CM, Aguilar A, Boshoff K (2020). Consumer involvement in university education programs in the nursing, midwifery, and allied health professions: a systematic scoping review. Int J Nurs Stud.

[10] Bennett-Weston A, Gay S, Anderson ES (2022). A theoretical systematic review of patient involvement in health and social care education. Adv Heal Sci Educ.

[11] Robinson K, Webber M (2013). Models and effectiveness of service user and carer involvement in social work education: a literature review. Br J Soc Work.

[12] Ferri P, Rovesti S, Padula MS, D’Amico R, Di Lorenzo R (2019). Effect of expert-patient teaching on empathy in nursing students: a randomized controlled trial. Psychol Res Behav Manag.

[13] Spencer J, Godolphin W, Karpenko N, Towle A. Can patients be teachers? Involving patients and service users in healthcare professionals’ education. 2011. https://www.health.org.uk/sites/default/files/CanPatientsBeTeachers.pdf.

[14] Rhodes CA, Nyawata ID (2011). Service user and carer involvement in student nurse selection: key stakeholder perspectives. Nurse Educ Today.

[15] Codsi MP, Karazivan P, Rouly G, Leclaire M, Boivin A (2021). Changing relationships: how does patient involvement transform professional identity? An ethnographic study. BMJ Open.

[16] Oermann MH, Reynolds SS, Granger BB (2022). Using an implementation science framework to advance the science of nursing education. J Prof Nurs.

[17] Higginbottom GMA, Pillay JJ, Boadu NY (2013). Guidance on performing focused ethnographies with an emphasis on healthcare research. Qual Rep.

[18] Ryan CL, Cant R, McAllister MM, Vanderburg R, Batty C (2022). Transformative learning theory applications in health professional and nursing education: an umbrella review. Nurse Educ Today.

[19] Robinson S, Shumar W (2014). Ethnographic evaluation of entrepreneurship education in higher education; a methodological conceptualization. Int J Manag Educ.

[20] Gertner AK, Franklin J, Roth I, Cruden GH, Haley AD, Finley EP, Hamilton AB, Palinkas LA, Powell BJ (2021). A scoping review of the use of ethnographic approaches in implementation research and recommendations for reporting. Implement Res Pract.

[21] Padula MS, Lui F, Ferri P, Draghetti L., Barbi S, Rossi F, Portera D. Imparare la medicina con l’insegnamento dei pazienti. 2020. In: 12° Rapporto sulla condizione assistenziale dei malati oncologici. [Learning medicine with the teaching of patients. 2020. In: 12th Report on the welfare condition of cancer patients] https://osservatorio.favo.it/dodicesimo-rapporto/parte-quinta/imparare-medicina/.

[22] Holmes AGD (2020). Researcher positionality - A consideration of its influence and place in qualitative research - a new researcher guide. Shanlax Int J Educ.

[23] Olmos-Vega FM, Stalmeijer RE, Varpio L, Kahlke R (2022). A practical guide to reflexivity in qualitative research: AMEE Guide 149. Med Teach.

[24] Cigliuti K. Cosa sono questi «appunti alla buona dall’aria innocente»? La costruzione delle note etnografiche. [What are these ‘casual notes with an innocent air’? The construction of ethnographic notes]. Firenze Univ Press; 2014. p. 93–125. https://media.fupress.com/files/pdf/24/2824/2824_6988.

[25] Robinson SG (2013). The relevancy of ethnography to nursing research. Nurs Sci Q.

[26] Ghirotto L, De Panfilis L, Di Leo S (2020). Health professionals learning qualitative research in their workplace: a focused ethnography. BMC Med Educ.

[27] Teo KJH, Teo MYK, Pisupati A, Ong RS, Goh CK, Seah CH, Toh YR, Burla N, Koh NS, Tay KT, Ong YT (2022). Assessing professional identity formation (PIF) amongst medical students in Oncology and Palliative Medicine postings: a SEBA guided scoping review. BMC Palliat Care.

[28] Lim JY, Ong SYK, Ng CYH, Chan KL, Wu SY, So WZ, Tey GJ, Lam YX, Gao NL, Lim YX, Tay RY (2023). A systematic scoping review of reflective writing in medical education. BMC Med Educ.

[29] Charon R (2001). Narrative medicine. Ann Intern Med.

[30] Chu SY, Wen CC, Lin CW (2020). A qualitative study of clinical narrative competence of medical personnel. BMC Med Educ.

[31] Sarraf-Yazdi S, Teo YN, How AEH, Teo YH, Goh S, Kow CS, Lam WY, Wong RS, Ghazali HZ, Lauw SK, Tan JR (2021). A scoping review of professional identity formation in undergraduate medical education. J Gen Intern Med.

[32] Mitchell KM, Roberts T, Blanchard L (2021). Reflective writing pedagogies in action: a qualitative systematic review. Int J Nurs Educ Scholarsh.

[33] Bjerkvik LK, Hilli Y (2019). Reflective writing in undergraduate clinical nursing education: a literature review. Nurse Educ Pract.

[34] Velasco J, Sanmartín FJ, Gálvez-Lara M, Cuadrado F, Moriana JA. Psychological effects of professional exposure to trauma and human suffering: systematic review and meta-analysis. Trauma Violence Abus. 2022;152483802210743. 10.1177/15248380221074314. 10.1177/15248380221074314 35202557

[35] Haycock-Stuart E, Donaghy E, Darbyshire C (2016). Involving users and carers in the assessment of preregistration nursing students’ clinical nursing practice: a strategy for patient empowerment and quality improvement?. Mol Ecol.

[36] Speed S, Griffiths J, Horne M, Keeley P (2012). Pitfalls, perils and payments: service user, carers and teaching staff perceptions of the barriers to involvement in nursing education. Nurse Educ Today.

[37] Riessman CK, Holstein JA, Gubrium JF (2002). Analysis of personal narratives. Insider interviewing. New lenses, new concerns.

[38] D’Cruz K, Douglas J, Serry T (2020). Sharing stories of lived experience: a qualitative analysis of the intersection of experiences between storytellers with acquired brain injury and storytelling facilitators. Br J Occup Ther.

[39] Mezirow J (1981). A critical theory of adult learning and education. Adult Educ.

[40] Happell B, Bennetts W (2016). Triumph and adversity: exploring the complexities of consumer storytelling in mental health nursing education. Int J Ment Health Nurs.

[41] McMahon-Parkes K, Chapman L, James J (2016). The views of patients, mentors and adult field nursing students on patients’ participation in student nurse assessment in practice. Nurse Educ Pract.

[42] Unwin P, Rooney J, Cole C (2018). Service user and carer involvement in students’ classroom learning in higher education. J Furth High Educ.

[43] Reitmaier A, Davies S, Reveling Smith L, Mangan-Danckwart D, Hongerholt K, Klinkner J (2015). Discovering intergenerativity: an evaluation of learning partnerships between student nurses and older adults. Int J Older People Nurs.

[44] Feijoo-Cid M, García‐Sierra R, García R, Ponce Luz H, Fernández‐Cano MI, Portell M (2022). Transformative learning experience among nursing students with patients acting as teachers: mixed methods, non‐randomized, single‐arm study. J Adv Nurs.

[45] Happell B, Waks S, Bocking J, Horgan A, Manning F, Greaney S, Goodwin J, Scholz B, van der Vaart KJ, Allon J, Hals E (2019). I felt some prejudice in the back of my head: nursing students’ perspectives on learning about mental health from experts by experience. J Psychiatr Ment Health Nurs.

[46] Guraya SY, Barr H (2018). The effectiveness of interprofessional education in healthcare: a systematic review and meta-analysis. Kaohsiung J Med Sci.

[47] Sy MP, Panotes A, Cho D, Pineda RC, Martin P (2022). A rapid review of the factors that influence service user involvement in interprofessional education, practice, and research. Int J Environ Res Public Health.

[48] Ackerson LK, Viswanath K (2009). The social context of interpersonal communication and health. J Health Commun.

[49] Towle A, Godolphin W (2013). Patients as educators: interprofessional learning for patient-centred care. Med Teach.

[50] Solomon P (2011). Student perspectives on patient educators as facilitators of interprofessional education. Med Teach.

[51] Andreassen P, Christensen MK, Møller JE (2020). Focused ethnography as an approach in medical education research. Med Educ.

