# Supplementary material for: Learning to teach with patients and caregivers: a focused ethnography
Source: BMC Med Educ. 2024 Mar 3;24:224. doi: 10.1186/s12909-024-05197-5 (PMC10910666; doi:10.1186/s12909-024-05197-5)
Supplement: Supplementary file 1 — Additional file 1. SyllabusT. his file contains the first draft of the syllabus of the post-graduate course ‘Didactic methodology for teaching with patients and caregivers as teachers’. [file 12909_2024_5197_MOESM1_ESM.docx]

**Additional File 1. Syllabus of the post-graduate course ‘Didactic methodology for teaching with patients and caregivers as teachers’**

| Learning Outcomes  Learning to teach in partnership to humanise care is the main learning outcome for all participants.  Particularly, patients and caregivers will learn how to teach together with healthcare professionals and to transmit their experiential knowledge of living with disease to students.  Healthcare professionals will learn how to integrate patients’ and caregivers’ stories into the lessons and work in collaboration with them. | | | | |
| --- | --- | --- | --- | --- |
| Module | **Title and Content** | **Objectives** | **Teaching Methodology** | **Hours** |
| Module I | Introduction: Course objectives, programme, and content.  Healthcare Professionals’ Degree Curricula: What content can be integrated with patients' life experiences? | Participants will know what it means to involve patients and caregivers and how patients' life experiences can be integrated in HPE | Frontal instruction | 3 |
| Module II | Sickness and healing in literature and humanistic science. | The participants will gain an understanding of the synergy between the humanities and social sciences and biomedical disciplines to provide an "integrated approach" to the experience of illness and care. | Film and frontal instruction | 6 |
| Module III | Narration is learning: From listening to and analysing patients' illness narratives to the discovery of experiential content to be learned in medicine. | Participants will become acquainted with narrative medicine and will be able to listen to and analyze patients' illness narratives to discover experiential content relevant to medicine. | Narration and groupwork | 11 |
| Module IV | Techniques and methods of the ‘lesson-in-tandem’. | Healthcare professionals, patients, and caregivers will be able to plan and conduct lessons in partnership. | Groupwork and simulation of ‘lesson-in-tandem’ | 11 |
| Module V | Interdisciplinarity and interprofessionalism as values in patients’ integrated care pathways. | The participants will gain knowledge and awareness of the importance of interdisciplinarity and interprofessionalism as values in integrated care pathways for patients. | Groupwork and ‘lessons-in-tandem’ | 11 |
