# Supplementary material for: Learning to teach with patients and caregivers: a focused ethnography
Source: BMC Med Educ. 2024 Mar 3;24:224. doi: 10.1186/s12909-024-05197-5 (PMC10910666; doi:10.1186/s12909-024-05197-5)
Supplement: Supplementary file 2 — Additional file 2. Interview guide. This file contains the first draft of the semi-structured interview guiding questions. [file 12909_2024_5197_MOESM2_ESM.docx]

**Additional File 2. Interview guide**

Step 1. INTRODUCTION

- Introduce yourself
- Ask the participant if s/he has read and signed the submitted documents and consent forms and if s/he has any questions about the study.
- Recall the rationale and purpose of the study and ask if the participant has any questions.
- Inform the participant of how the interview will be conducted, remind him/her that the interview will be recorded, and ensure him/her the data will remain anonymous.
- Mark the date and time and start recording.

Step 2. INTERVIEW

- Could you tell me about your experience in the post-graduate course ‘Didactic methodology for teaching with patients and caregivers as teachers’?
- Why did you take this course?
- During the course, participants were asked to write and share their narrations. Can you tell me about your narration? How did you feel about it?
- Thinking about an activity or episode that has particularly impressed you, what comes to mind?
- What did you take from this experience?
- How do you evaluate the teaching methods used? Comparing the activities carried out in presence and online, what aspects would you highlight?
- Thinking about the class, how was relating to the group?
- How did you feel?
- Is there anything that struck you about it?
- How did you experience the relationship with the patients and caregivers as teachers?

Step 3. CLOSING

- Ask the participant if s/he has any comments to add.
- Thank the participant for the interview and remind him/her that s/he will be contacted for a subsequent interview and could be contacted again during the analysis and discussion of the results.
