# Supplementary material for: Learning to teach with patients and caregivers: a focused ethnography
Source: BMC Med Educ. 2024 Mar 3;24:224. doi: 10.1186/s12909-024-05197-5 (PMC10910666; doi:10.1186/s12909-024-05197-5)
Supplement: Supplementary file 3 — Additional file 3. Focus Group guide. This file contains the first draft of the focus group guide. [file 12909_2024_5197_MOESM3_ESM.docx]

**Additional File 3. Focus group guide**

Step 1. OPENING

- Moderator and assistant presentation.
- Clarification of the topic and purpose of the research.
- Rules definition and registration consent request.
- What is expected with this focus group.

Step 2. DISCUSSION

- Thinking about the course with the patient-teacher, what has remained with you about the experience? How does this resonate today?
- Can you tell me what you think you needed the course to teach the patient-teacher?
- What have you learned?
- What do you think you need to complete the lesson-in-tandem? Is it implementable? How?
- Within the group, there were active and multiple communicative exchanges facilitated by different informal moments, which strengthened relationships between participants. During the course, there was a change in roles. Many of you have recognised yourself as patient- or caregiver-teachers, while others have taken on different educational group management roles. How do you characterise yourself now within your group? How do you see the group today?
- Are there any aspects that you would like to discuss together or other issues that have come to mind that you want to share in this focus group?

Step 3. CLOSING

- Thank participants for their commitment and the time given to this research.
- Summarizes the main topics that emerged from the discussion.
- Ask if all agree on the main issues that emerged.
- Ask them to rank the issues that emerged (to be re-evaluated).
